# Comparative Efficacy of ^177^Lu and ^90^Y for Anti-CD20 Pretargeted Radioimmunotherapy in Murine Lymphoma Xenograft Models

**DOI:** 10.1371/journal.pone.0120561

**Published:** 2015-03-18

**Authors:** Sofia H. L. Frost, Shani L. Frayo, Brian W. Miller, Johnnie J. Orozco, Garrett C. Booth, Mark D. Hylarides, Yukang Lin, Damian J. Green, Ajay K. Gopal, John M. Pagel, Tom A. Bäck, Darrell R. Fisher, Oliver W. Press

**Affiliations:** 1 Clinical Research Division, Fred Hutchinson Cancer Research Center, Seattle, WA, United States of America; 2 Pacific Northwest National Laboratory, Richland, WA, United States of America; 3 College of Optical Sciences, The University of Arizona, Tucson, AZ, United States of America; 4 Departments of Medicine and Bioengineering, University of Washington, Seattle, WA, United States of America; 5 Sahlgrenska Academy, University of Gothenburg, Gothenburg, Sweden; 6 Dade Moeller Health Group, Richland, WA, United States of America; Department of Medical Lab Technology, Faculty of Applied Medical Sciences, Taibah University, SAUDI ARABIA

## Abstract

**Purpose:**

Pretargeted radioimmunotherapy (PRIT) is a multi-step method of selectively delivering high doses of radiotherapy to tumor cells while minimizing exposure to surrounding tissues. Yttrium-90 (^90^Y) and lutetium-177 (^177^Lu) are two of the most promising beta-particle emitting radionuclides used for radioimmunotherapy, which despite having similar chemistries differ distinctly in terms of radiophysical features. These differences may have important consequences for the absorbed dose to tumors and normal organs. Whereas ^90^Y has been successfully applied in a number of preclinical and clinical radioimmunotherapy settings, there have been few published pretargeting studies with ^177^Lu. We therefore compared the therapeutic potential of targeting either ^90^Y or ^177^Lu to human B-cell lymphoma xenografts in mice.

**Methods:**

Parallel experiments evaluating the biodistribution, imaging, dosimetry, therapeutic efficacy, and toxicity were performed in female athymic nude mice bearing either Ramos (Burkitt lymphoma) or Granta (mantle cell lymphoma) xenografts, utilizing an anti-CD20 antibody-streptavidin conjugate (1F5-SA) and an ^90^Y- or ^177^Lu-labeled 1,4,7,10-tetraazacyclododecane-1,4,7,10-tetraacetic acid (DOTA)-biotin second step reagent.

**Results:**

The two radionuclides displayed comparable biodistributions in tumors and normal organs; however, the absorbed radiation dose delivered to tumor was more than twice as high for ^90^Y (1.3 Gy/MBq) as for ^177^Lu (0.6 Gy/MBq). More importantly, therapy with ^90^Y-DOTA-biotin was dramatically more effective than with ^177^Lu-DOTA-biotin, with 100% of Ramos xenograft-bearing mice cured with 37 MBq ^90^Y, whereas 0% were cured using identical amounts of ^177^Lu-DOTA-biotin. Similar results were observed in mice bearing Granta xenografts, with 80% of the mice cured with ^90^Y-PRIT and 0% cured with ^177^Lu-PRIT. Toxicities were comparable with both isotopes.

**Conclusion:**

^90^Y was therapeutically superior to ^177^Lu for streptavidin-biotin PRIT approaches in these human lymphoma xenograft models.

## Introduction

Non-Hodgkin lymphoma (NHL) is the tenth most common cancer worldwide, and although improved therapies have led to increased survival rates the malignancy caused over 199,000 deaths in 2012 [1]. In the United States the standard treatment for B-cell NHL is chemotherapy combined with rituximab, a chimeric anti-CD20 monoclonal antibody (MAb), but approximately 40% of the patients still die of this disease, emphasizing the desirability of improved therapeutic approaches. Radioimmunotherapy (RIT) is a modality that may provide a greater therapeutic window than chemotherapy, i.e. equally or more effective treatment with milder side effects. Two radiolabeled MAbs have been FDA-approved for treatment of follicular and transformed NHL [2, 3], but the success of RIT has nevertheless been hampered by suboptimal pharmacokinetics of the high-molecular weight radioimmunoconjugates. To improve the radioactive dose distribution within tumors and the ratio of radionuclide deposited in tumors compared with non-malignant tissues, a number of multi-step, pretargeted radioimmunotherapy (PRIT) regimens have been proposed [4, 5]. One of the most strongly validated methods utilizes the extremely high affinity between biotin and streptavidin (SA), enabling improved tumor-to-normal organ absorbed dose ratios [6, 7].

For CD20-expressing lymphomas, dramatically enhanced efficacy and safety has been shown in a series of studies comparing PRIT with single-step RIT, predominantly using ^90^Y-labeled 1,4,7,10-tetraazacyclododecane-1,4,7,10-tetraacetic acid (DOTA)-biotin for delivery of radioactivity to MAb-SA pretargeted tumors [8–12]. The encouraging results obtained with ^90^Y-PRIT have inspired researchers to explore other radioactive elements that could improve the outcome even further. For NHL, radionuclides with physical characteristics resembling those of ^131^I are particularly interesting because of the therapeutic relevance of this isotope demonstrated in several clinical anti-CD20 RIT studies [13–15]; however, adequate methods for labeling DOTA-biotin with ^131^I are lacking. Instead, the similarities with ^131^I in terms of half-life and radiation energy together with the chemical features shared with ^90^Y have highlighted the radiolanthanide ^177^Lu as highly promising for targeted therapy [16–18]. Table 1 summarizes the radiophysical properties of ^131^I, ^90^Y, and ^177^Lu. ^90^Y is a pure beta emitter, which necessitates using a gamma emitting surrogate (^111^In) for scintillation imaging in clinical trials. Conversely, ^177^Lu emits gamma rays with moderate energy and low, yet sufficient, abundance for direct imaging, avoiding the radiation exposure of healthcare personnel associated with the high-energy gamma emitter ^131^I. Other theoretical advantages of ^177^Lu over ^90^Y for PRIT include decreased damage to non-target tissues owing to the shorter beta particle range, and the potential for reduced marrow toxicity coupled with increased energy deposition to tumors because of the better match between physical half-life and biological retention half-time of the radiolabeled construct in blood and target tissues. ^177^Lu can be stably and effectively incorporated into the DOTA-biotin macrocycle through well-established chelation methods developed for ^90^Y.

**Table 1:** Properties of selected therapeutic beta emitters.

Isotope	Half-life (d)	β (MeV)		γ^a^ (keV)	Max range (mm)
		Max	Avg		
^131^I	8.0	0.60	0.18	364 (82), 637 (6.5)	2.0
^90^Y	2.7	2.28	0.94	–	11.0
^177^Lu	6.7	0.50	0.13	208 (11), 113 (7)	1.5

^a^Percentages in parentheses

The aim of this study was to assess the potential use of ^177^Lu-DOTA-biotin for anti-CD20 MAb-SA PRIT side-by-side with the corresponding ^90^Y-PRIT regimen, through comparison of tumor-to-normal organ absorbed dose ratios, cure rates, survival curves, and treatment-related side effects. We report here comparative biodistribution, imaging, therapy, and toxicity experiments in athymic mice bearing either Burkitt or mantle cell human lymphoma xenografts. These murine models of NHL are well-characterized and thoroughly validated as preclinical tools for studying principles of radiobiology and pharmacology prior to human trials [19]. The toxicities were similar and *in vivo* distributions showed comparable uptakes of ^177^Lu and ^90^Y in all studied organs and tissues. However, due to the different emission characteristics associated with the two radionuclides, the mean absorbed dose to tumor was more than twice as high for ^90^Y as for ^177^Lu after administration of the same level of radioactivity, leading to a dramatic difference in survival favoring ^90^Y-PRIT. These data demonstrate that ^90^Y is the preferred beta-particle emitting radionuclide for PRIT in human lymphoma xenograft models.

## Materials and Methods

### Cell lines

The human Ramos [20] (Burkitt lymphoma) and Granta-519 [21] (mantle cell lymphoma) lines were obtained from American Type Culture Collection (ATCC; Bethesda, MD). All cells were maintained in RPMI 1640 medium (Gibco) supplemented with 10% defined fetal bovine serum (HyClone), penicillin (100 U/mL), and streptomycin (100 μg/mL) in a 5% carbon dioxide incubator. Cell viability was greater than 95% by trypan blue exclusion for all experiments described.

### Antibodies, streptavidin-antibody conjugates, and clearing agent

Murine monoclonal antibody 1F5 (anti-CD20) was produced in the MAb production facility at Fred Hutchinson Cancer Research Center (FHCRC; Seattle, WA) from its hybridoma (gift from Clay Siegall; Seattle Genetics, Seattle, WA) using a hollow fiber bioreactor system. The HB8181 MAb (anti-HSV-1 glycoprotein) was produced by injecting its hybridoma (ATCC) into pristane-primed mice to generate ascites. The MAb was subsequently purified from the ascitic fluid by protein G immunoadsorption column chromatography. The covalent chemical conjugates 1F5-SA and HB8181-SA were prepared as previously described [8]. A synthetic clearing agent (CA) containing 16 *N*-acetyl-galactosamine residues and one biotin moiety per dendrimeric molecule (NAGB; Aletheon Pharmaceuticals) was used to effectively direct excess circulating MAb-SA from the bloodstream to the liver for clearance by utilizing hepatic galactose receptors [6].

### ^177^Lu/^90^Y labeling of DOTA-biotin

The bifunctional DOTA-biotin ligand was synthesized as previously described [22]. Radiolabeling with either ^177^Lu or ^90^Y was performed in a metal-free environment. Up to 20 μL of ^177^LuCl_3_ (27–65 mCi) or ^90^YCl_3_ (15–72 mCi) in 0.05M HCl (PerkinElmer) was diluted with 0.5 M ammonium acetate, pH 5.3, to a total volume of 200 μL. To this solution was added 60 μg (5.0 μL) of aqueous DOTA-biotin (12 mg/mL) followed by aqueous sodium ascorbate (500 mg/mL) to a final ascorbate concentration of 50 mg/mL. The reaction mixture was heated and maintained at 83°C for 45 min. After cooling to room temperature, DTPA in aqueous solution (100 mM) was added to a final DTPA concentration of 10 mM, to chelate any unbound ^90^Y or ^177^Lu. ^90^Y-DTPA and ^177^Lu-DTPA are rapidly excreted via the kidneys, thus minimizing non-target accumulation of free radionuclide. The labeling efficiencies were typically greater than 97%, as determined by binding of radiolabeled DOTA-biotin to immobilized avidin beads.

### Mouse studies

All mouse experiments were carried out in accordance with the recommendations in the Guide for the Care and Use of Laboratory Animals of the National Institutes of Health, and all efforts were made to minimize suffering. Animals were maintained under protocols approved by the Fred Hutchinson Cancer Research Center Institutional Animal Care and Use Committee (FHCRC IACUC); this study was authorized specifically under IACUC file No. 1490. Female athymic nude mice (Harlan Sprague Dawley) aged 6–8 weeks were injected subcutaneously with 1×10^7^ Ramos or Granta cells in the right flank to generate solid human lymphoma xenografts. To facilitate synchronous, uniform establishment of tumors, 4 μg of anti-asialo-GM1 antiserum (Wako Chemicals USA) was injected intraperitoneally (i.p.) on day—1 before inoculation, followed by another injection on day 4 and then weekly until the end of the study. All mice were provided with a biotin-deficient diet (Harlan Teklad) from 5–6 days before to 6 days after injection of MAb-SA conjugates. Euthanasia was performed through carbon dioxide overexposure per the American Veterinary Medical Association (AVMA) guidelines.

### Biodistribution and dosimetry

Mice with palpable Ramos xenografts (diameter ~8 mm) were selected for *in vivo* distribution studies and injected intravenously (i.v.) with 1.4 nmol (300 μg) of either a specific anti-CD20 conjugate (1F5-SA) or a non-specific isotype-matched IgG2a analog (HB8181-SA). The conjugated antibodies were injected in combination with 400 μg of unmodified HB8181 to decrease non-specific binding of MAb-SA to Fc receptors in spleen, liver, and bone marrow. Twenty hours later, excess circulating first step MAb-SA was removed from the bloodstream by i.v. administration of 5.8 nmol (50 μg) of NAGB. The radiolabeled second step molecule, 2.4 nmol (2 μg) of either ^177^Lu- or ^90^Y-labeled DOTA-biotin, was injected i.v. 24 hours after the first step. Tissues (blood, lung, liver, spleen, stomach, kidneys, small intestine, large intestine, muscle, femur, tail, residual carcass, and tumor) were subsequently harvested 4, 24, 48, or 120 hours after the radio-DOTA-biotin injection (p.i.) with five mice per treatment group and time point. All samples were weighed and measured for radioactivity using a Packard Cobra II Auto Gamma counter, and the percent injected activity per gram (%IA/g) ± standard deviation calculated with corrections for decay and background. Radiation absorbed doses were calculated for circulating blood and for each excised organ, tissue, and tumor based on wet tissue weights and time-activity plots that were constructed from the biodistribution data. To determine the total number of radioactive transformations (decays) in each tissue, we plotted %IA/g (not decay-corrected) versus time for each organ or tissue, and then employed curve fitting software (i.e.,TableCurve2D, Systat Software Inc.; CurveExpert, Daniel G. Hyams) to obtain a best-fit single- or double-exponential function by linear least-squares regression analysis to the time-activity data. Each best-fit function was integrated from time zero to infinity to estimate the total decays taking place in the organ or tissue per unit administered activity (MBq-hours per MBq). If the data were not well-correlated to a single- or double- exponential, a more appropriate function was obtained and integrated, or the area-under-curve was obtained by linear splines and trapezoidal integration; long-term retention was estimated by exponential fitting to the last two data points and extrapolating to infinity. The total number of decays were multiplied by the energy emitted from ^90^Y or ^177^Lu per decay, using a method that accounts for the size, shape, and anatomic placement of organs and tissues in the mouse, absorbed energy fractions, and cross-organ contributions to dose from incorporated ^90^Y or ^177^Lu [23]. Absorbed fractions for ^177^Lu were obtained from Miller et al., who extended the work of Hui et al. to include additional isotopes [24]. Standard dosimetry principles were then applied to calculate mean radiation absorbed doses to each organ or tissue.

In addition, images of cryosections (thickness 10 μm) of kidney, liver, and tumor were taken 24 hours p.i. to assess the radioactivity distribution using the iQID camera, a novel single-particle digital autoradiography imager that enables activity quantification of alpha and beta emitters in excised tissues [25]. The instrument is composed of an image intensifier, a lens, and a CCD/CMOS camera. Radioactive samples are applied to a scintillation screen (DRZ-Std; MCI Optonix) that is placed directly onto the camera surface, and as the beta particles interact with the scintillator optical photons are emitted and imaged onto the camera sensor, generating a real-time digital autoradiograph with high spatial resolution. In the current study, consecutive cryosections of all imaged tissues were cut and transferred to glass slides. The slides were formalin-fixed and stained with hematoxylin and eosin (H&E) to enable histological comparison with the intra-organ radioactivity distribution displayed in the iQID images.

### Therapy

The therapeutic efficacies of the two PRIT regimens were compared in groups of ten Ramos or Granta-xenografted mice per treatment group. The animals were injected with 300 μg of either 1F5-SA or HB8181-SA, together with 400 μg of HB8181, followed 20 hours later by 50 μg of NAGB. Radio-DOTA-biotin (2 μg) was administered four hours after the clearing agent; the amount of DOTA-biotin was the same for all mice, irrespective of administered radioactivity. In the Ramos model three radioactivity levels were used per radionuclide: 15, 30, or 37 MBq; for Granta only the optimal amount determined from the prior Ramos experiments, 37 MBq of ^177^Lu- or ^90^Y-DOTA-biotin, was studied. In addition, ten tumor-bearing mice that received no treatment (PBS only) served as a control group for each study. All injections were performed i.v. and a biotin-free diet was provided as described above. Mice were weighed and tumor dimensions (length, width, and height) measured three times a week, either until mice were euthanized due to treatment-related toxicity (body weight ≤ 70% of baseline or poor general status) or tumor burden (tumor volume ≥ 1200 mm^3^), or until tumors had completely disappeared (complete remission) in all mice. Weekly examinations were then performed until the end of study. Tumor volumes were calculated using the ellipsoid formula: V (mm^3^) = 4/3π × (length/2) × (width/2) × (height/2). To decrease radiation-induced weight loss, all mice were provided with nutrient fortified water gel (DietGel Recovery; Clear H20) and injected s.c. with 1 mL of 0.9% sodium chloride solution after weighing until no mouse in any group was below 80% of their initial body weight.

### Toxicity

In order to assess for possible long-term radiation-induced side effects from the two PRIT regimens, a study was designed using three different treatment groups of ten mice each receiving 37 MBq ^177^Lu-DOTA-biotin, 37 MBq ^90^Y-DOTA-biotin, or no treatment (control). Tumor-free mice were used to enable long-term follow-up. All injections were performed according to the therapy protocol previously described. Retro-orbital blood sampling was performed for complete blood counts (CBC; 20 μL/mouse) and assessment of renal and hepatic function (100 μL/mouse). Baseline values were taken eight days before the injection of radio-DOTA-biotin, after which CBC measurements were performed on day 1, 3, 5, 7, 9, 14, 21, then weekly for two months, and then bi-weekly until the end of the study (day 183). Blood sampling for serum chemistries was performed on a weekly schedule. The status of the animals was evaluated in terms of body weight, hematocrit, white blood cell (WBC) count, and platelet (PLT) count, in addition to serum hepatic enzymes (alkaline phosphatase, alanine aminotransferase, and aspartate aminotransferase) and renal function tests (blood urea nitrogen and creatinine).

## Results

### Biodistribution and dosimetry

**Fig 1:**
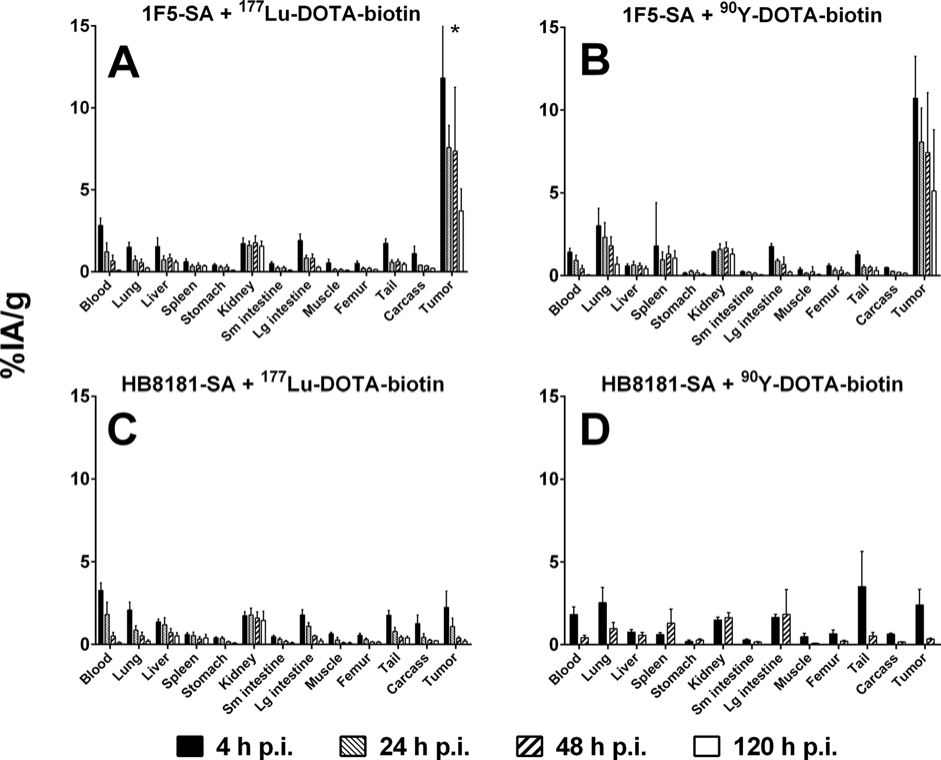
Comparative biodistributions of radioactivity in mice bearing subcutaneous Ramos xenografts. Mice were injected with 1.4 nmol 1F5-SA (A and B) or HB8181-SA (C and D), together with 400 μg of HB8181. Circulating MAb-SA was cleared by administration of 5.8 nmol of NAGB 20 hours p.i. Four hours later, 2.4 nmol of either ^177^Lu- (A and C) or ^90^Y-labeled (B and D) DOTA-biotin was injected. Tissues were harvested 4, 24, 48, or 120 hours p.i. and the radioactive content expressed as %IA/g (± SD; n = 5) after corrections for decay and background. *Error bar extends to 19.4%IA/g.

The *in vivo* distributions of radiolabeled DOTA-biotin pretargeted with either 1F5-SA or HB8181-SA are shown in Fig. 1. The accumulation of labeled DOTA-biotin in tumors pretargeted with 1F5-SA was high by 4 hours p.i. (11.8 ± 7.6%IA/g for ^177^Lu; 10.7 ± 2.5%IA/g for ^90^Y), whereas much lower tumor uptakes were observed in groups administered the non-specific MAb-SA conjugate (2.2 ± 1.0%IA/g for ^177^Lu; 2.4 ± 1.0%IA/g for ^90^Y). Forty-eight hours after injection, less than 0.5%IA/g was detected in tumors pretargeted with HB8181-SA for either radionuclide. Conversely, 1F5-SA-pretargeted tumors retained 7.4 ± 3.9%IA/g of ^177^Lu and 7.4 ± 3.6%IA/g of ^90^Y at the same time point. Even after 120 hours, 3.7 ± 1.3%IA/g of ^177^Lu and 5.1 ± 3.7%IA/g of ^90^Y remained in tumors pretargeted with 1F5-SA. No statistically significant differences in tumor uptake were seen between the two radioisotopes, for any of the studied time points (Holm-Sidak test; p = 0.77, 0.67, 0.98, and 0.45 for 4, 24, 48, and 120 hours p.i., respectively). Comparison of specific and non-specific pretargeting agents demonstrated significantly higher tumor uptake for all studied time points using 1F5-SA (Holm-Sidak test; ^177^Lu: p = 0.02 for 4 h p.i., p < 0.01 for 24, 48 and 120 h p.i.; ^90^Y: p < 0.01 for 4 and 48 h p.i.). Blood clearance was rapid and the activity concentrations generally low in all non-target tissues, without apparent dependence on radioisotope or MAb-SA specificity.

Table 2 lists the mean absorbed doses calculated using the acquired biodistribution data, assuming uniform intra-organ distributions of radioactivity. The applied dosimetry model extends the MIRD concept of cross-organ dose in humans to the small dimensions of mouse tissues, taking into account beta energy specific absorbed fractions and organ mass. The absorbed dose to tumor was 23.0 Gy/37 MBq for ^177^Lu-DOTA-biotin, with kidneys displaying the highest normal tissue dose (10.2 Gy/37 MBq). The resulting tumor-to-normal tissue dose ratios (TNTDR) were 9.4 and 2.3 for blood and kidneys, respectively. For ^90^Y-DOTA-biotin the corresponding doses were 49.7 and 12.9 Gy/37 MBq to tumor and kidneys with TNTDR of 12.4 and 3.9 for blood and kidneys, respectively.

**Table 2:** Mean radiation absorbed doses from radiolabeled DOTA-biotin in Ramos-xenografted mice pretargeted with 1F5-SA.

	^177^Lu-DOTA-Biotin			^90^Y-DOTA-Biotin		
Tissue	Gy/MBq	Gy/37 MBq	TNTDR^a^	Gy/MBq	Gy/37 MBq	TNTDR^a^
Blood	0.066	2.4	9.4	0.108	4.0	12.4
Lung	0.043	1.6	14.5	0.213	7.9	6.3
Liver	0.072	2.7	8.6	0.180	6.7	7.5
Spleen	0.043	1.6	14.5	0.154	5.7	8.7
Stomach	0.023	0.9	27.0	0.037	1.4	36.3
Kidney	0.275	10.2	2.3	0.348	12.9	3.9
Small intestine	0.019	0.7	32.7	0.039	1.4	34.4
Large intestine	0.060	2.2	10.4	0.161	6.0	8.3
Muscle	0.011	0.4	56.5	0.056	2.1	24.0
Femur	0.015	0.6	41.5	0.028	1.0	48.0
Tail	0.039	1.4	15.9	0.036	1.3	37.3
Carcass	0.027	1.0	23.0	0.069	2.6	19.5
Tumor	0.622	23.0		1.343	49.7	

^a^TNTDR = Tumor-to-normal tissue dose ratio

n = 5 per treatment and time point (4, 24, 48, and 120 hours p.i.)

### Imaging

**Fig 2:**
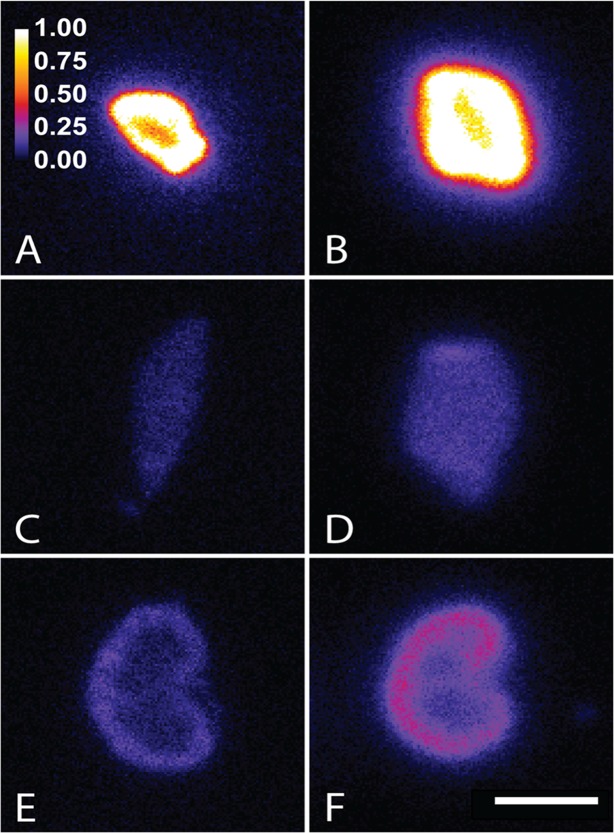
Autoradiographs acquired using the novel iQID camera. Panels show the distribution of ^177^Lu (A, C, and E) and ^90^Y (B, D, and F) 24 hours after injection of labeled DOTA-biotin in 1F5-SA-pretargeted tumor (A and B), liver (C and D), and kidney (E and F) cryosections. The scales apply to all panels; the color bar represents relative pixel intensity normalized to the mean for the tumor sections and the white scale bar indicates 5 mm.

The iQID autoradiographs corresponded well with the biodistribution data, confirming efficient tumor targeting and analogous sub-organ activity distributions for both radionuclides in tumor, liver, and kidney. Fig. 2 shows examples of intra-organ activity distributions from two mice 24 hours p.i., treated with either ^90^Y-DOTA-biotin or ^177^Lu-DOTA-biotin. To quantify the uniformity of the activity distributions, regions of interest were drawn around areas of high (“hot”), average, and low (“cold”) intensity, and the mean pixel values within those regions compared for each tissue (n = 3 per radionuclide). Tumor uptakes were heterogeneous, with on average 2.0 times higher signal in peripheral tumor regions compared with central regions and 1.2 times higher signal in peripheral regions compared with the mean for the whole tumor section in ^177^Lu-treated mice. The corresponding ratios for ^90^Y were 1.7 and 1.3. A correlation between the location of “hot” areas and necrotic regions was observed in sequential H&E stained tumor sections, indicating cell kill in targeted regions. For kidneys, the signal in the renal cortex was on average 2.2 times higher than that of the medulla and 1.4 times higher than the section mean for ^177^Lu; for ^90^Y the corresponding numbers were 1.9 and 1.3. The liver sections exhibited anticipated homogeneous activity profiles.

### Therapy

**Fig 3:**
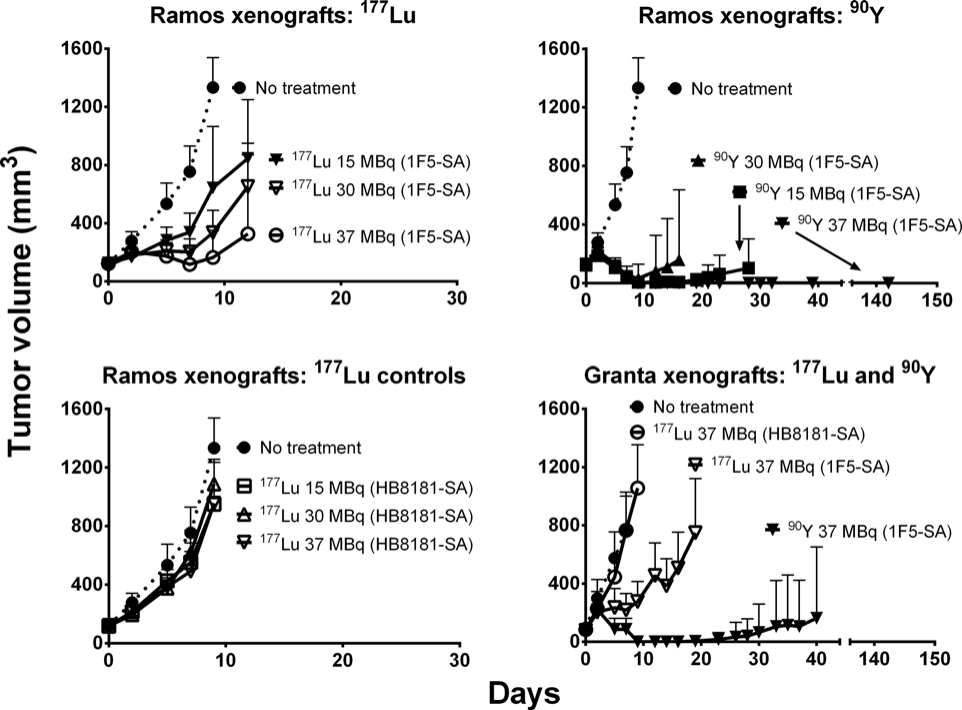
Regression of Ramos and Granta xenografts in athymic mice after either ^177^Lu- or ^90^Y-PRIT. Mice were injected with 1.4 nmol of 1F5-SA or HB8181-SA, together with 400 μg of HB8181. Circulating MAb-SA was cleared by administration of 5.8 nmol of NAGB after 20 hours, followed by 15–37 MBq of either ^177^Lu- or ^90^Y-labeled DOTA-biotin (2.4 nmol) 4 hours later. Ten xenograft mice per tumor model served as control group (PBS only). The curves were truncated at the time of euthanasia of the first mouse in each treatment group (mm^3^ ± SD; n = 10).

The mean tumor sizes at the start of therapy were 122 ± 6 mm^3^ for Ramos xenografts and 85 ± 8 mm^3^ for Granta tumors (mean ± SD). All control mice exhibited exponential tumor growth and were euthanized by day 12, regardless of protocol (untreated or non-specifically pretargeted) or xenograft (Ramos or Granta). Fig. 3 depicts the tumor volumes as a function of time after therapy for all treatment groups, demonstrating a clear difference in efficacy between ^177^Lu- and ^90^Y-PRIT. None of the administered ^177^Lu activities (15–37 MBq) were sufficient for achieving complete remission, although tumor growth was delayed in a dose-dependent manner. In contrast, the highest activity administered for ^90^Y (37 MBq) resulted in complete remissions in all mice bearing either Ramos (10 of 10 mice) or Granta (10 of 10) tumors, although one of the ten Granta-bearing animals exhibited a relapse 19 days after treatment.

**Fig 4:**
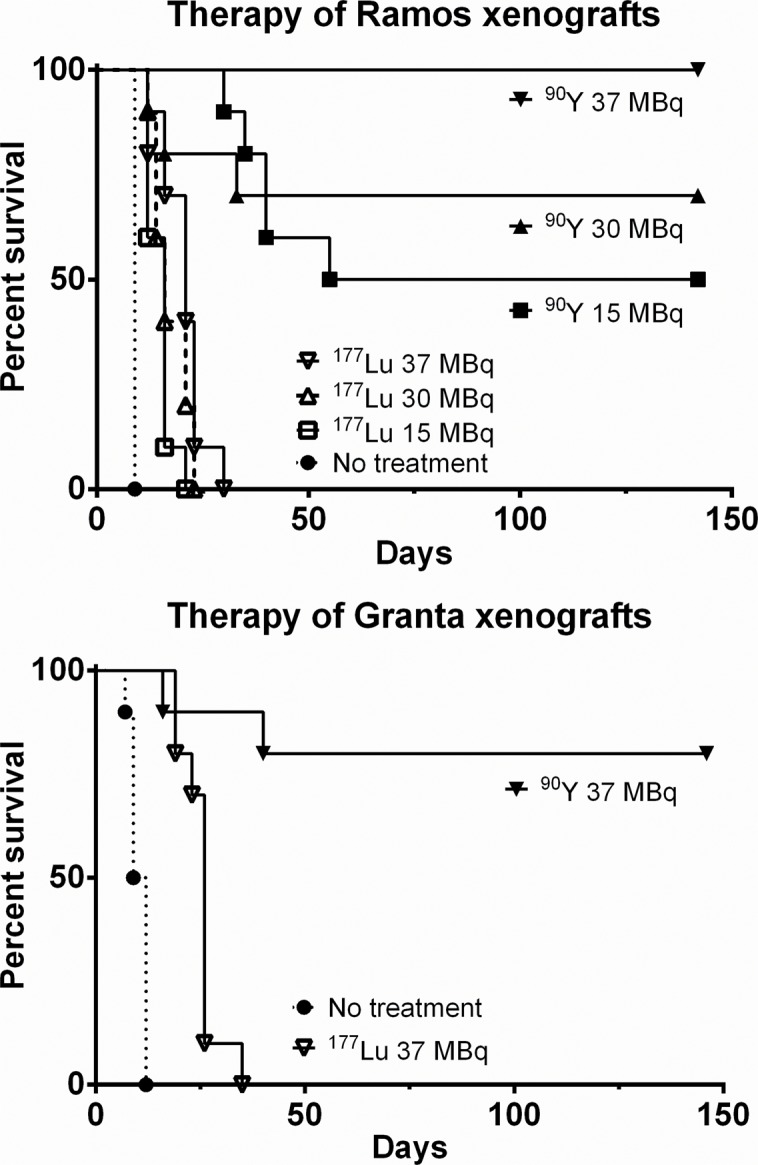
Kaplan-Meier survival curves of mice carrying either Ramos or Granta xenografts after ^177^Lu-/ ^90^Y-PRIT. Athymic nude mice were i.v. injected with 300 μg of 1F5-SA or HB8181-SA, together with 400 μg of HB8181, followed 20 hours later by 50 μg of NAGB. Four hours later, ^177^Lu- or ^90^Y-labeled DOTA-biotin was administered: 15, 30, or 37 MBq for Ramos xenografts; 37 MBq for Granta (n = 10). Control groups received no treatment (PBS only).

The resulting Kaplan-Meier survival curves are shown in Fig. 4, demonstrating clear superiority of ^90^Y compared to ^177^Lu in this pretargeted setting. All treatments using ^177^Lu-DOTA-biotin resulted in 0% long-term survival, regardless of the tumor model. Two of the 1F5-SA-pretargeted mice treated with 37 MBq ^177^Lu died of toxicity; one from weight loss and poor general condition on day 14, and the other from intra-abdominal hemorrhage on day 19. All other ^177^Lu-treated mice were eventually euthanized due to disease progression to comply with IACUC guidelines. Among animals treated with 37 MBq ^90^Y-DOTA-biotin survival was 10/10 for Ramos xenografts and 8/10 for Granta 20 weeks after treatment; one Granta xenograft mouse was euthanized on day 16 due to weight loss and another on day 40 because of recurring disease after complete remission. Of the Ramos-xenografted ^90^Y-irradiated mice, one animal treated with 30 MBq ^90^Y-DOTA-biotin was sacrificed due to weight loss; all other non-survivors were euthanized due to disease progression. Remaining mice were followed for 142–146 days, after which they were sacrificed without exhibiting any evident signs of disease or treatment-related adverse effects.

### Toxicity

**Fig 5:**
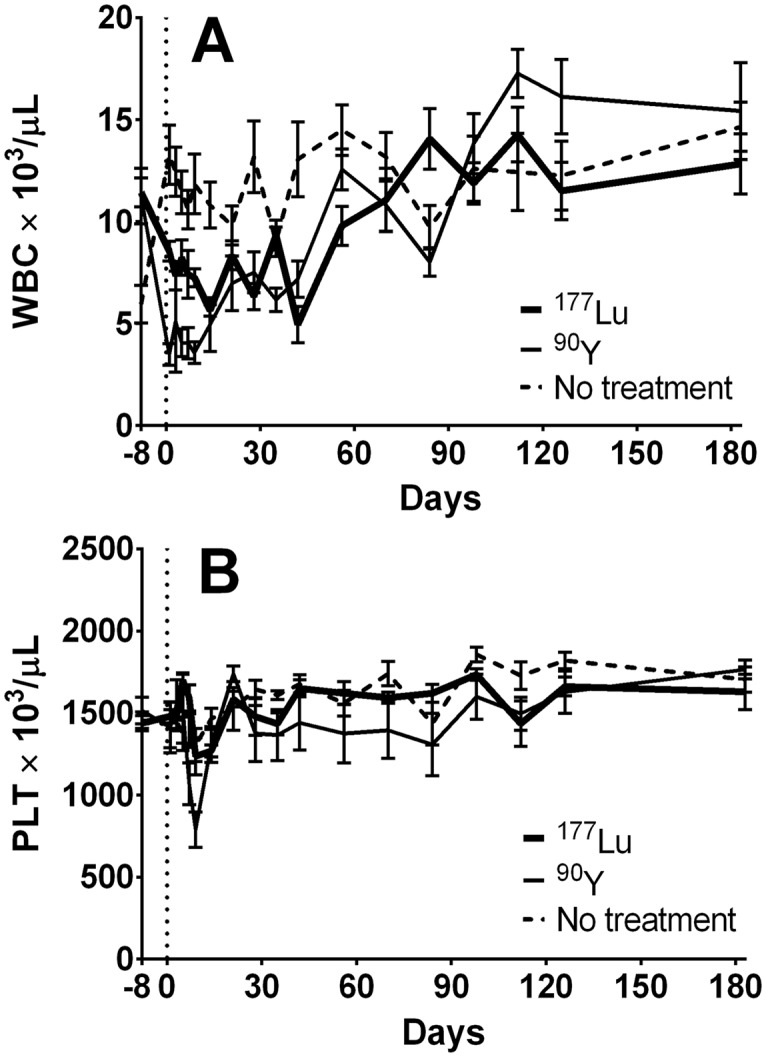
Radiation-induced toxicity in non-xenografted mice after treatment with 37 MBq of ^177^Lu- or ^90^Y-PRIT. 1F5-SA (300 μg) was co-injected with 400 μg of HB8181, followed 20 hours later by 50 μg of NAGB. Four hours later, 37 MBq of either ^177^Lu- or ^90^Y-labeled DOTA-biotin was administered. The control group received no injections. (WBC (A) and PLT (B) counts ± SD; n = 10).

Long term toxicity studies were conducted in mice not bearing tumor xenografts because all xenografted mice receiving ^177^Lu-PRIT at any dose (and all xenografted mice receiving no treatment), succumbed to disease progression well before long-term adverse effects could be evaluated. Acute radiation-induced toxicity appeared milder in non-tumor bearing mice, with no significant weight loss or anemia following PRIT with 37 MBq of either ^177^Lu or ^90^Y, possibly due to the lack of retention of radioactivity in tumors. As shown in Fig. 5, the WBC counts dropped for both treatment groups with a more pronounced effect in ^90^Y-irradiated mice (t-test; p < 0.01), with average leukocyte counts reaching their nadir of 31% of the baseline value by the day after treatment. Eight weeks later, the average WBC count in the ^90^Y-irradiated group was comparable to the average value in the untreated control group (p = 0.02). For ^177^Lu-PRIT the radiotoxic effect was similar to that of ^90^Y-PRIT, albeit delayed, reaching a nadir of 43% of baseline six weeks after irradiation. The WBC count did not recover fully until week ten after treatment. The difference in mean WBC counts at their respective nadirs was not statistically significant for the two treatments (t-test; p = 0.17), and neither was the average time to WBC recovery (t-test; p = 0.56). The ^90^Y-treated group exhibited mild thrombocytopenia with PLT counts of approximately 800 × 10^3^/μL on day 9, but recovered to 1700 × 10^3^/μL by day 21. A minor effect on PLT counts was also seen in the ^177^Lu group, with a nadir (1200 × 10^3^/μL) on day 9 and recovery (1600 × 10^3^/μL) by day 21. The difference in thrombocytopenia was not statistically significant between the two treatments (Holm-Sidak test; p = 0.01 for day 9). No significant differences were detected in serum hepatic enzyme levels or renal function between treated and untreated mice, regardless of radionuclide.

## Discussion

The clinical potential of ^177^Lu for use in targeted therapies has been considered for over 20 years, based on promising radiophysical properties, robustness of chelation methods, and availability. Half-life, decay chain, range, and energy deposition are emission characteristics that not only influence the likelihood of tumor eradication and toxic side-effects of a given radionuclide treatment, but also have logistical consequences for patients and determine the shielding requirements for protection of medical personnel. RIT optimization must account for all these parameters, and the combination of low-energy beta emissions, photons suitable for clinical *in vivo* radiolocalization, and a 6.7-day half-life has highlighted ^177^Lu as a promising candidate. A number of regimens have been explored for targeted ^177^Lu-treatment of colon carcinoma [26], various adenocarcinomas [27], ovarian carcinoma [28, 29], lung cancer [30], neuroendocrine tumors [31], HER-2 expressing tumors [32], and gastric cancer [33] among others. Previous RIT studies for lymphomas have mainly employed the commonly used isotopes, ^131^I or ^90^Y. Some authors have speculated that the nature of this disease may warrant the use of a radionuclide with a shorter path length to more efficiently irradiate the diffusely infiltrating tumor cells and broaden the therapeutic window [34]. Forrer et al. recently demonstrated the therapeutic potential of ^177^Lu-DOTA-rituximab with manageable hematologic toxicity in a dose-escalation study involving 31 patients with various relapsed B-cell lymphomas [35]. Clinical responses were observed in all patient groups and at all dose levels (although some very brief), and out of 13 follicular lymphoma patients 8 were alive after a median of 84 months.

A few preclinical ^177^Lu-PRIT studies have been conducted aiming for improvement of the suboptimal pharmacokinetics associated with single-step RIT. Two main approaches have been evaluated, targeting human colon tumor xenografts: i) anti-histamine-succinyl-glycine (HSG) bispecific antibodies (bsMAb) in combination with ^177^Lu-labeled HSG-containing peptides [36, 37], and ii) CC49-SA fusion proteins and ^177^Lu-DOTA-biotin [16, 18, 38]. Sharkey et al. compared radiation dose estimates for the ^90^Y- or ^177^Lu-labeled HSG-peptide IMP-241 after bsMAb pretargeting of GW-39 xenografts, concluding that although ^90^Y delivers a higher dose to the tumors per administered MBq, at equal absorbed doses to kidneys ^177^Lu-IMP-241 and ^90^Y-IMP-241 would potentially enable equal tumor doses [36]. Delayed LS174T tumor growth and prolonged survival was achieved by Schoffelen et al. through repeated treatment cycles using a humanized recombinant anti-HSG bsMAb and the labeled di-HSG peptide ^177^Lu-IMP288, with mild toxicity [37]. Lewis et al., Buchsbaum et al. and Mohsin et al. all explored SA-biotin PRIT for treatment of LS174T colorectal tumors in nude mice. Buchsbaum et al. used an intraperitoneal model, resulting in prolonged survival after either ^90^Y- or ^177^Lu-PRIT [38]. However, in their regional model 30 MBq (800 μCi) of ^90^Y-DOTA-biotin caused 60% early deaths from toxicity, whereas the corresponding ^177^Lu-treatment produced no similar effect. Lewis et al. studied the dosimetry after association of ^149^Pm-, ^166^Ho-, and ^177^Lu-DOTA-biotin to CC49 scFvSA-pretargeted xenografts [16], and Mohsin et al. followed up by comparing the therapeutic efficacies of single-step and pretargeted RIT using the three radiolanthanides [18]. ^149^Pm- and ^177^Lu-PRIT resulted in identical tumor growth delay, whereas ^166^Ho-PRIT was less successful. For single-step RIT ^177^Lu was more efficient. Six months after PRIT, survival rates were 20, 0, and 20% for ^149^Pm, ^166^Ho, and ^177^Lu, respectively; for single-step RIT the corresponding numbers were 0, 10, and 12.5%, and the authors designated ^177^Lu as the optimal radiolanthanide in this tumor model.

In contrast to our expectations and theoretical projections, the results presented here clearly demonstrate the superiority of ^90^Y compared to ^177^Lu as the radionuclide for incorporation into the second step reagent in pretargeted treatment regimens targeting CD20-expressing human lymphoma xenografts in athymic mice. The maximum activity administered in the dose-escalating therapy study was 37 MBq, a level that caused lethal toxicity in 2/10 ^177^Lu-DOTA-biotin-treated Ramos xenograft mice, despite intensive supportive care (saline injections and nutrition gel). The corresponding number for ^90^Y-treated mice was 0/10, although 1/10 mice treated with 30 MBq ^90^Y-DOTA-biotin was euthanized due to weight loss. For Granta xenografts the numbers were slightly different; no toxicity-related deaths were caused by 37 MBq ^177^Lu-DOTA-biotin, whereas 37 MBq of ^90^Y-DOTA-biotin resulted in one. This indicates that 37 MBq was very close to the maximum tolerated activity for either radionuclide, and was thus designated the optimum for these models. Considering the absorbed doses to normal organs, it may still be argued that ^177^Lu-PRIT might allow administration of slightly higher activity than ^90^Y-PRIT, but the increase is not likely large enough to outweigh the considerably lower energy deposition in tumors resulting from the radiophysical differences. Despite nearly identical biodistributions, the absorbed dose from ^90^Y to tumors was more than twice that of ^177^Lu, primarily relating to differences in energy emitted per decay and fraction of energy emitted by beta versus gamma decay. According to Miller et al., the absorbed fraction of monoenergetic electrons to tumors of ~150 mg is approximately 0.5 for ^90^Y and 0.9 for ^177^Lu [24]. Because the equilibrium dose constant of ^177^Lu is 1/6 of that of ^90^Y [39], the activity required for equivalent dose to tumor is three to six times higher for ^177^Lu compared to ^90^Y; an unrealistic order of increase in these models. Furthermore, the proven pharmacokinetic efficiency of this pretargeting system diminishes the influence of benefits attributed to the longer half-life of ^177^Lu. For RIT regimens in which radioimmunoconjugates circulate for an extended time before localizing at tumor sites the decay rate of ^90^Y becomes problematic in terms of radiation-induced toxicity, but when high tumor-to-normal-tissue dose ratios can be achieved as early as four hours p.i., as in these studies, the theoretical benefits of ^177^Lu are clearly overshadowed by the disadvantages.

Another determinant of therapeutic efficacy is the intratumoral absorbed dose distribution. Inefficient diffusion of radiolabeled vectors and antigen expression heterogeneity are factors that decrease the radiation coverage, enabling tumors to re-establish from unharmed malignant cells. The longer beta particle range for ^90^Y *in vivo* compensates for “cold” regions, resulting in a more uniform dose delivery [40]. The iQID-autoradiographs revealed non-uniform distributions of both radionuclides in 1F5-SA-pretargeted tumors, the signal in high-activity regions being approximately twice of that in low-activity regions. With an average tumor diameter of ~8 mm at the time of irradiation, the 1.5-mm maximum path length of ^177^Lu was presumably inferior to that of ^90^Y (11.0 mm) in making up for this observed heterogeneity.

In conclusion, our results convincingly show that ^90^Y was superior to ^177^Lu for streptavidin-biotin PRIT in the studied murine lymphoma xenograft models. This finding has been used for selecting ^90^Y as the radionuclide for an upcoming Phase I trial of anti-CD20 PRIT in patients with relapsed B-cell lymphomas.

## Supporting Information

S1 ARRIVE Checklist A completed ARRIVE (Animal Research: Reporting of *In Vivo* Experiments) checklist describing the reported animal studies. (PDF)
